# Essential amino acids as diagnostic biomarkers of hepatocellular carcinoma based on metabolic analysis

**DOI:** 10.18632/oncotarget.28306

**Published:** 2022-11-22

**Authors:** Yuji Morine, Tohru Utsunomiya, Hisami Yamanaka-Okumura, Yu Saito, Shinichiro Yamada, Tetsuya Ikemoto, Satoru Imura, Shohei Kinoshita, Akiyoshi Hirayama, Yasuhito Tanaka, Mitsuo Shimada

**Affiliations:** ^1^Department of Surgery, Institute of Biomedical Sciences, Tokushima University Graduate School, Tokushima 770-8503, Japan; ^2^Department of Clinical Nutrition and Food Management, Institute of Biomedical Sciences, Tokushima University Graduate School, Tokushima 770-8503, Japan; ^3^Institute for Advanced Biosciences, Keio University, Tsuruoka, Yamagata 997-0052, Japan; ^4^Systems Biology Program, Graduate School of Media and Governance, Keio University, Fujisawa, Kanagawa 252-0882, Japan; ^5^Department of Gastroenterology and Hepatology, Faculty of Life Sciences, Kumamoto University, Kumamoto 860-8556, Japan

**Keywords:** metabolomics, essential amino acid, hepatocellular carcinoma, diagnostic biomarker

## Abstract

Metabolomics, defined as the comprehensive identification of all small metabolites in a biological sample, has the power to shed light on phenotypic changes associated with various diseases, including cancer. To discover potential metabolomic biomarkers of hepatocellular carcinoma (HCC), we investigated the metabolomes of tumor and non-tumor tissue in 20 patients with primary HCC using capillary electrophoresis–time-of-flight mass spectrometry. We also analyzed blood samples taken immediately before and 14 days after hepatectomy to identify associated changes in the serum metabolome. Marked changes were detected in the different quantity of 61 metabolites that could discriminate between HCC tumor and paired non-tumor tissue and additionally between HCC primary tumors and colorectal liver metastases. Among the 30 metabolites significantly upregulated in HCC tumors compared with non-tumor tissues, 10 were amino acids, and 7 were essential amino acids (leucine, valine, tryptophan, isoleucine, methionine, lysine, and phenylalanine). Similarly, the serum metabolomes of HCC patients before hepatectomy revealed a significant increase in 16 metabolites, including leucine, valine, and tryptophan. Our results reveal striking differences in the metabolomes of HCC tumor tissue compared with non-tumor tissue, and identify the essential amino acids leucine, valine, and tryptophan as potential metabolic biomarkers for HCC.

## INTRODUCTION

Hepatocellular carcinoma (HCC) is the most prevalent primary liver cancer and the third leading cause of cancer-related deaths worldwide [1, 2]. Despite advances in treatment modalities such as surgical techniques, radiofrequency ablation, and novel molecular targeted agents that include multi-kinase inhibitors (e.g., sorafenib and lenvatinib) and monoclonal antibodies (e.g., anti-PD-L1 [atezolizumab] and anti-vascular endothelial growth factor [bevacizumab]), the prognosis of HCC patients is still unacceptable. This is due in large part to the advanced stage of the disease at diagnosis resulting from its asymptomatic nature [3–7]. Post-treatment relapse of HCC, including multicentric recurrence, is also common, particularly among high-risk patients such as those with hepatitis B virus (HBV) or hepatitis C virus (HCV) infection, non-alcoholic fatty liver disease, and non-alcoholic steatohepatitis. Therefore, there is an urgent need to identify novel diagnostic biomarkers to assist in the early detection of HCC, while current diagnostic approaches for HCC consist of measurement of serum α-fetoprotein levels and imaging of liver parenchyma by Gd-EOB-DTPA-enhanced magnetic resonance imaging have been established [8–14].

Metabolomics, a relatively recently introduced ‘omics’ technology, is defined as the comprehensive analysis of small molecules (typically < 1.5 kDa), including amino acids, sugars, lipids, and inorganic ions, in a biological sample [15]. The human metabolome is estimated to consist of between 2500 and 8000 metabolites [16, 17]. Currently, the three main analytical methods for metabolome analysis are gas chromatography / mass spectrometry, liquid chromatography–mass spectrometry, and capillary electrophoresis–mass spectrometry, each of which analyzes a different class of metabolites [15, 18]. In the last two decades, these analytical methods have been advanced and optimized to enable disease phenotyping and biomarker identification, and they have been increasingly applied in clinical settings [19, 20]. We previously reported on the use of an advanced technique, capillary electrophoresis–time-of-flight mass spectrometry (CE-TOFMS), to monitor changes in liver metabolites following hepatectomy with ischemia reperfusion [21]. Several studies have investigated alterations in the metabolome during hepatocarcinogenesis and identified several candidate metabolic biomarkers that could potentially discriminate between liver diseases, including HCC [22–26]. However, those findings were various and a consensus decision on a reliable metabolomic biomarker of liver disease and/or hepatocarcinogenesis has not yet been reached.

In the present study, we sought to discover reliable metabolomic biomarkers for HCC diagnosis by elucidating metabolic alterations in primary HCC tumor tissue compared with non-tumor tissue, and additionally by investigating the serum metabolomic profiles before and after curative hepatectomy.

## RESULTS

### Metabolic alterations in HCC tumor tissue

**Figure 1:**
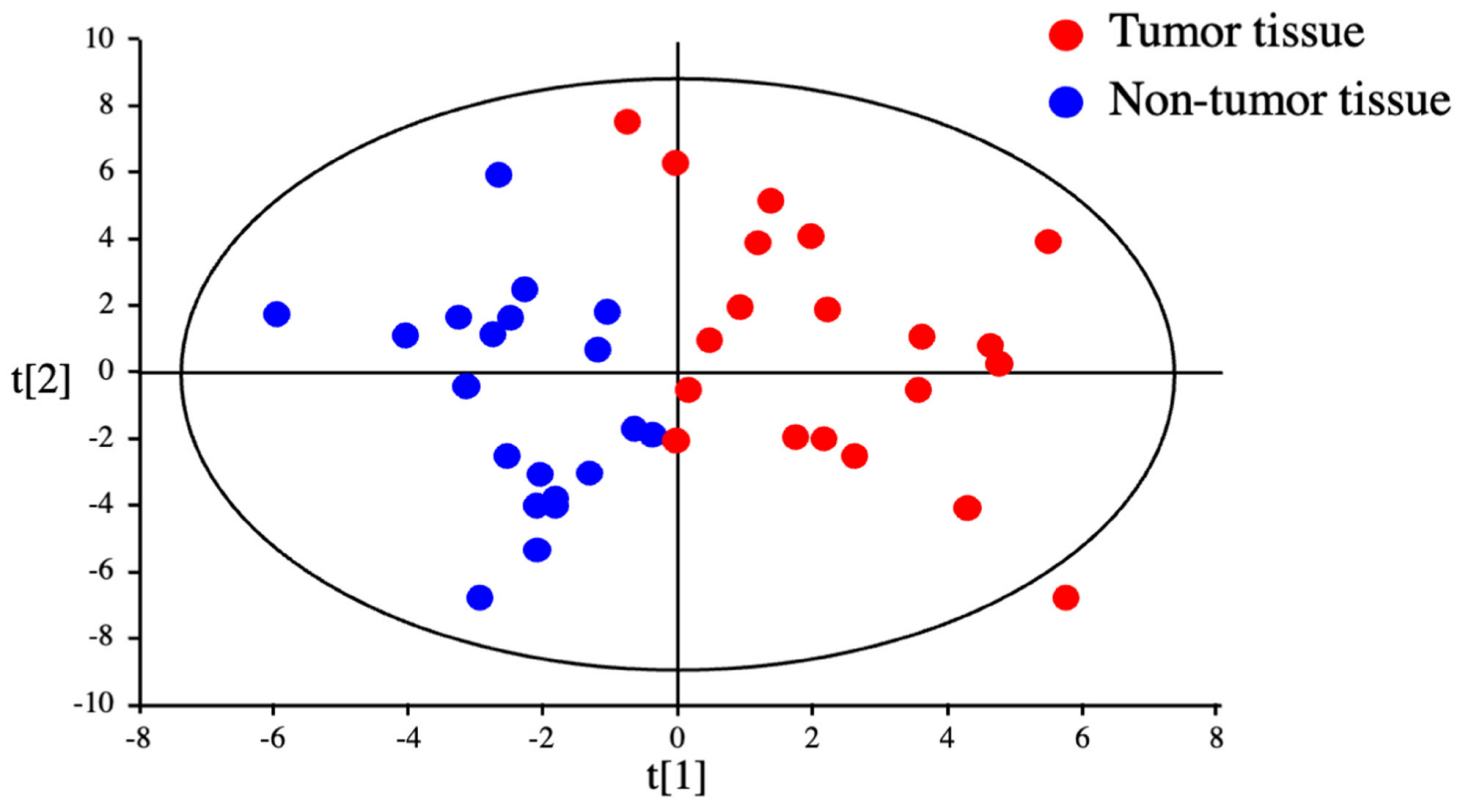
Discrimination between tumor and non-tumor tissues from HCC patients by metabolomics. OPLS-DA of HCC tumor and non-tumor tissues could clearly distinguished.

**Figure 2:**
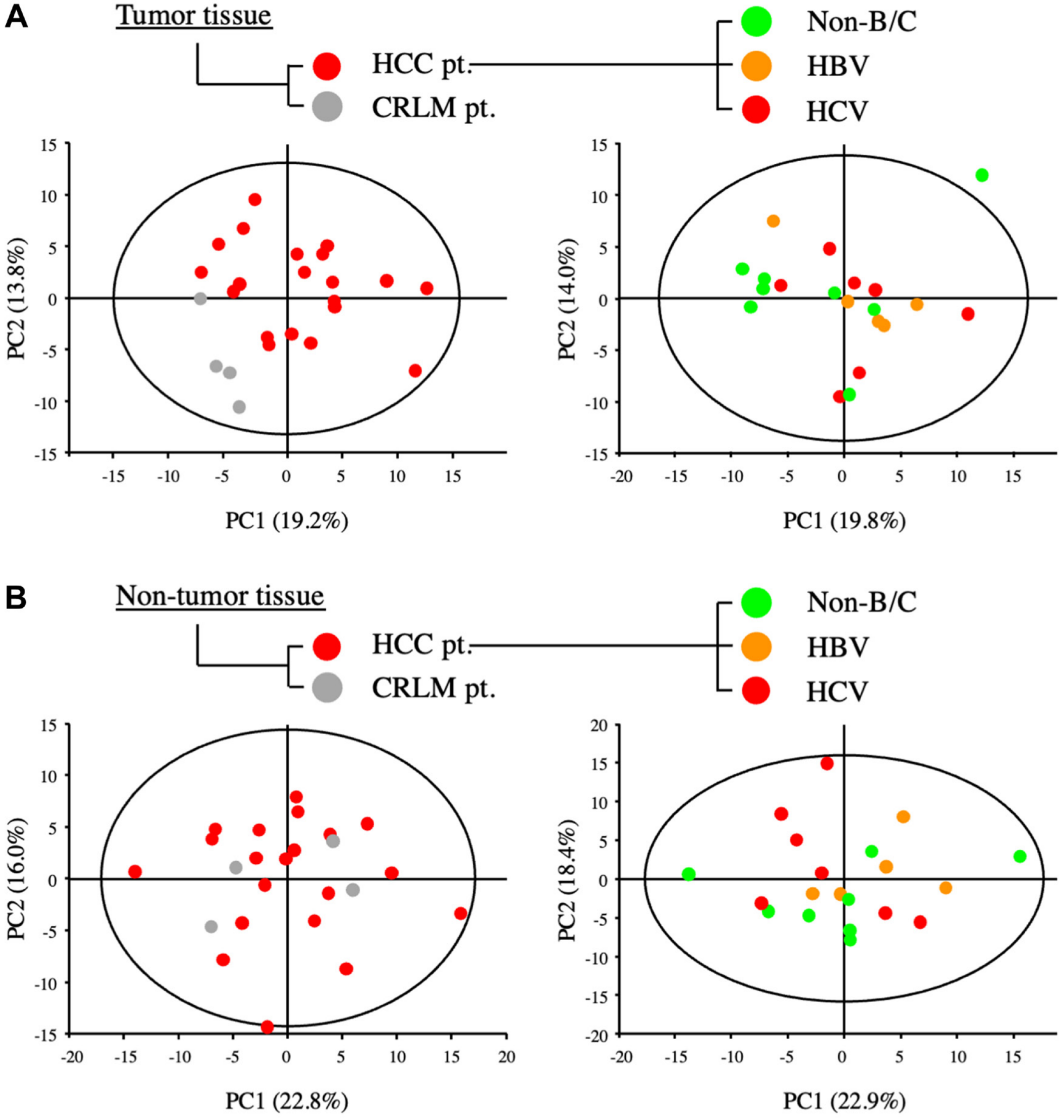
Metabolic analysis of primary and metastatic liver tumors from patients with HCC and colorectal cancer. (**A**) PCA of components of tumor tissues from patients with HCC (HCC-pt), CRLM (CLRM-pt), and HCC of viral (HBV, HCV) and non-viral etiology (non-B/C). (**B**) PCA of components of non-tumor liver tissues from patients as described for (A).

The metabolomic profiles of tumor and non-tumor liver tissues from 20 HCC patients and 4 CRLM patients were obtained and subjected to OPLS-DA. As shown in the scores plot in Figure 1, the metabolomes of HCC tumor and non-tumor tissues could be clearly distinguished. Similarly, PCA revealed obvious differences in the component profiles of the 20 HCC tumor and 4 CRLM tissues (Figure 2A). The analysis did not distinguish between the metabolomic profiles of HCC tumors from HBV-infected (*n* = 5), HCV-infected (*n* = 7), or uninfected (*n* = 8) patients (Figure 2A), indicating that the metabolomes did not differ significantly between HCC of differing etiologies. Notably, the profiles were also similar between non-tumor tissue from patients with HCC and CRLM (Figure 2B) and between non-tumor tissue from patients with HCC of differing etiologies (Figure 2B).

**Figure 3:**
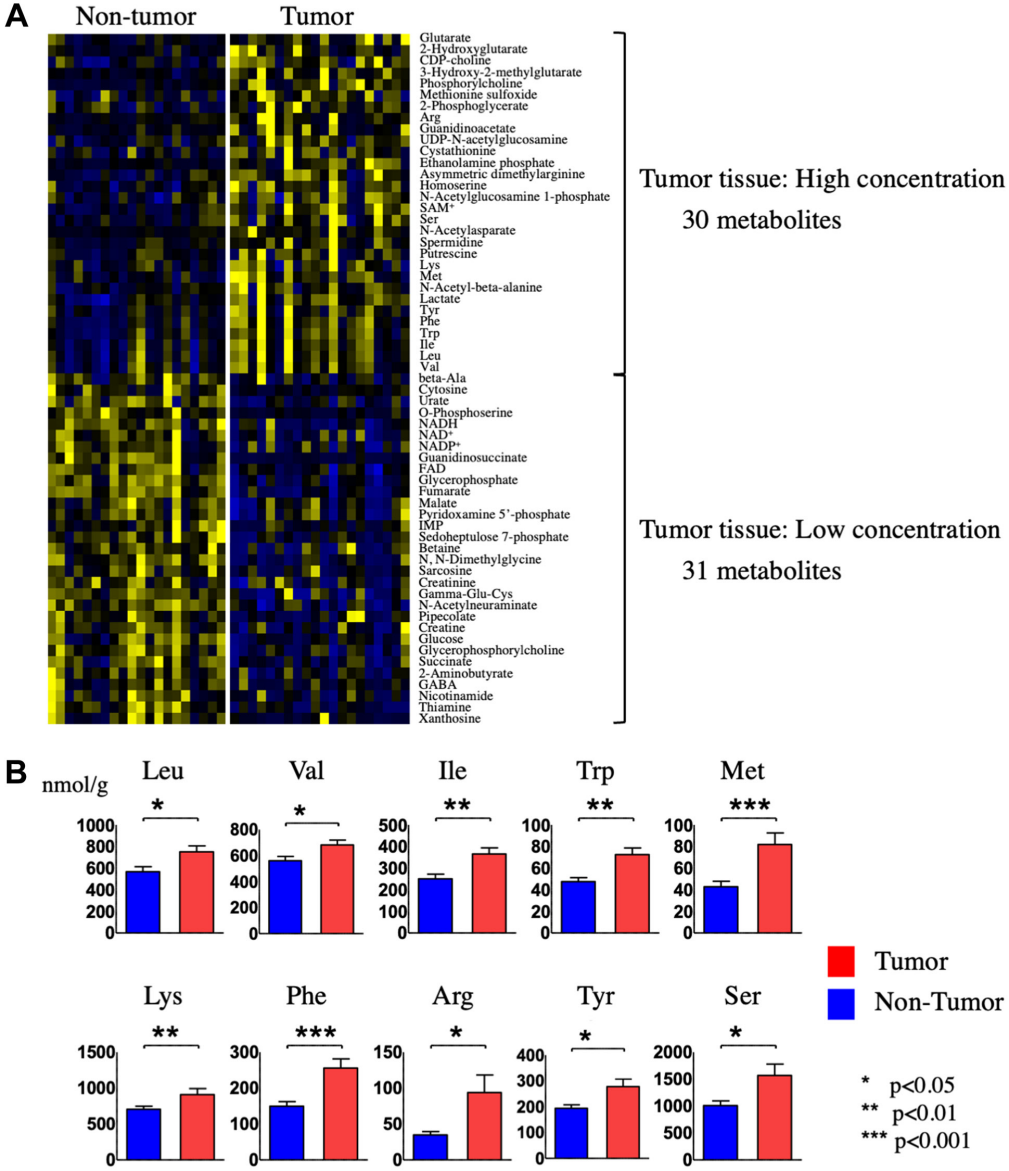
Identification and quantification of metabolites in tumor and non-tumor tissues from HCC patients. (**A**) Hierarchical clustering of metabolites present at different levels in HCC tumor tissue compared with non-tumor tissue. (**B**) Quantification of the indicated 10 amino acids in HCC tumor tissue compared with non-tumor tissue.

We analyzed a total of 179 metabolites in HCC tumor versus non-tumor tissues. Among these, 61 metabolites had a variable importance for projection (VIP) score of >1.0 and a *p* value < 0.05 and were thus considered critical metabolites for distinguishing between the metabolomic profiles of HCC tumor and non-tumor tissues (Supplementary Figure 1 and Table 1). Hierarchical clustering of the 61 metabolites revealed that 30 metabolites were significantly upregulated and 31 were significantly downregulated in HCC tumor tissue compared with non-tumor tissue (Figure 3A and Table 2). One of the metabolites upregulated in HCC tumor tissue was lactate, which is consistent with the known propensity of tumor cells to undergo elevated rates of aerobic glycolysis; a process known as the Warburg effect. In addition, 10 amino acids were increased in HCC tumor compared with non-tumor tissue, and of these, 7 were the essential amino acids (EAAs) valine, leucine, isoleucine, tryptophan, methionine, lysine, and phenylalanine (Figure 3B).

**Table 1:** Tissue metabolites that distinguish between HCC tumor tissue and non-tumor tissue (VIP >1.0, p < 0.05)

No.	Up-regulated metabolites in tumor tissue	VIP score	No.	Down-regulated metabolites in tumor tissue	VIP score
1	Lactate	1.78707	1	Pyridoxamine 5’-phosphate	2.4411
2	Phosphorylcholine	1.71512	2	Glycerophosphate	2.37857
3	Phe	1.65483	3	Guanidinosuccinate	2.17465
4	SAM^+^	1.63714	4	N-Acetylneuraminate	2.07864
5	Ethanolamine phosphate	1.62781	5	Thiamine	1.97634
6	Trp	1.56405	6	Urate	1.9265
7	CDP-choline	1.5565	7	N,N-Dimethylglycine	1.92546
8	3-Hydroxy-3-methylglutarate	1.51524	8	NADH	1.92425
9	Met	1.50306	9	FAD	1.84351
10	Cystathionine	1.48954	10	Glycerophosphorylcholine	1.79874
11	Ile	1.4859	11	Succinate	1.71503
12	Asymmetric dimethylarginine	1.37081	12	O-Phosphoserine	1.6733
13	N-Acetyl-beta-alanine	1.34241	13	Sedoheptulose 7-phosphate	1.62377
14	2-Hydroxyglutarate	1.28193	14	Glucose	1.61838
15	UDP-N-acetylglucosamine	1.2539	15	gamma-Glu-Cys	1.55918
16	Tyr	1.23994	16	2-Aminobutyrate	1.39563
17	Leu	1.21722	17	Cytosine	1.31766
18	Homoserine	1.20879	18	Sarcosine	1.28744
19	Guanidinoacetate	1.19845	19	NAD^+^	1.27527
20	Ser	1.19066	20	Xanthosine	1.26902
21	Val	1.18445	21	Nicotinamide	1.26782
22	N-Acetylaspartate	1.17003	22	Creatine	1.25463
23	Arg	1.1405	23	NADP^+^	1.24883
24	N-Acetylglucosamine 1-phosphate	1.12281	24	Betaine	1.18196
25	Spermidine	1.12021	25	Creatinine	1.12331
26	Glutarate	1.11995	26	IMP	1.1043
27	Methionine sulfoxide	1.11277	27	GABA	1.10039
28	Putrescine	1.07124	28	beta-Ala	1.09067
29	Lys	1.06571	29	Fumarate	1.09013
30	2-Phosphoglycerate	1.03023	30	Pipecolate	1.04196
			31	Malate	1.0326

**Table 2:** Serum metabolites that distinguish between HCC patients before and after hepatectomy (VIP >1.0, p < 0.05)

No.	Increased metabolites in post-Hx	VIP score	No.	Decrease metabolites in post-Hx	VIP score
1	Methionine sulfoxide	2.11817	1	His	2.30411
2	Glycolate	1.95728	2	Pentanoate + 3-Methylbutanoate	2.22692
3	N-Acetylglucosamine	1.79674	3	Val	2.07023
4	Asymmetric dimethylalginine	1.78249	4	Glycerophosphorylcholine	2.06643
5	Phe-Phe	1.59984	5	Trp	1.70211
6	Lactate	1.48632	6	Ala	1.66633
7	Hypoxanthine	1.48582	7	4-Oxohexanoate + 4-Acetylbutyrate	1.63735
8	beta-Ala	1.47965	8	Thr	1.63534
9	N-Acetylaspartate	1.42722	9	Carnitine	1.52112
10	N-Acetylglutamate	1.42277	10	4-Methyl-2-oxopentanoate	1.48188
11	3-Hydroxybutyrate	1.32702	11	3-Indoxyl sulfate	1.41297
12	Fumarate	1.2612	12	2-Oxoisopentanoate	1.37897
13	Allantoin	1.18354	13	Leu	1.36857
			14	Gly	1.36797
			15	Kynurenine	1.34456
			16	Azelate	1.14054

### Changes in serum metabolic profiles after hepatectomy

**Figure 4:**
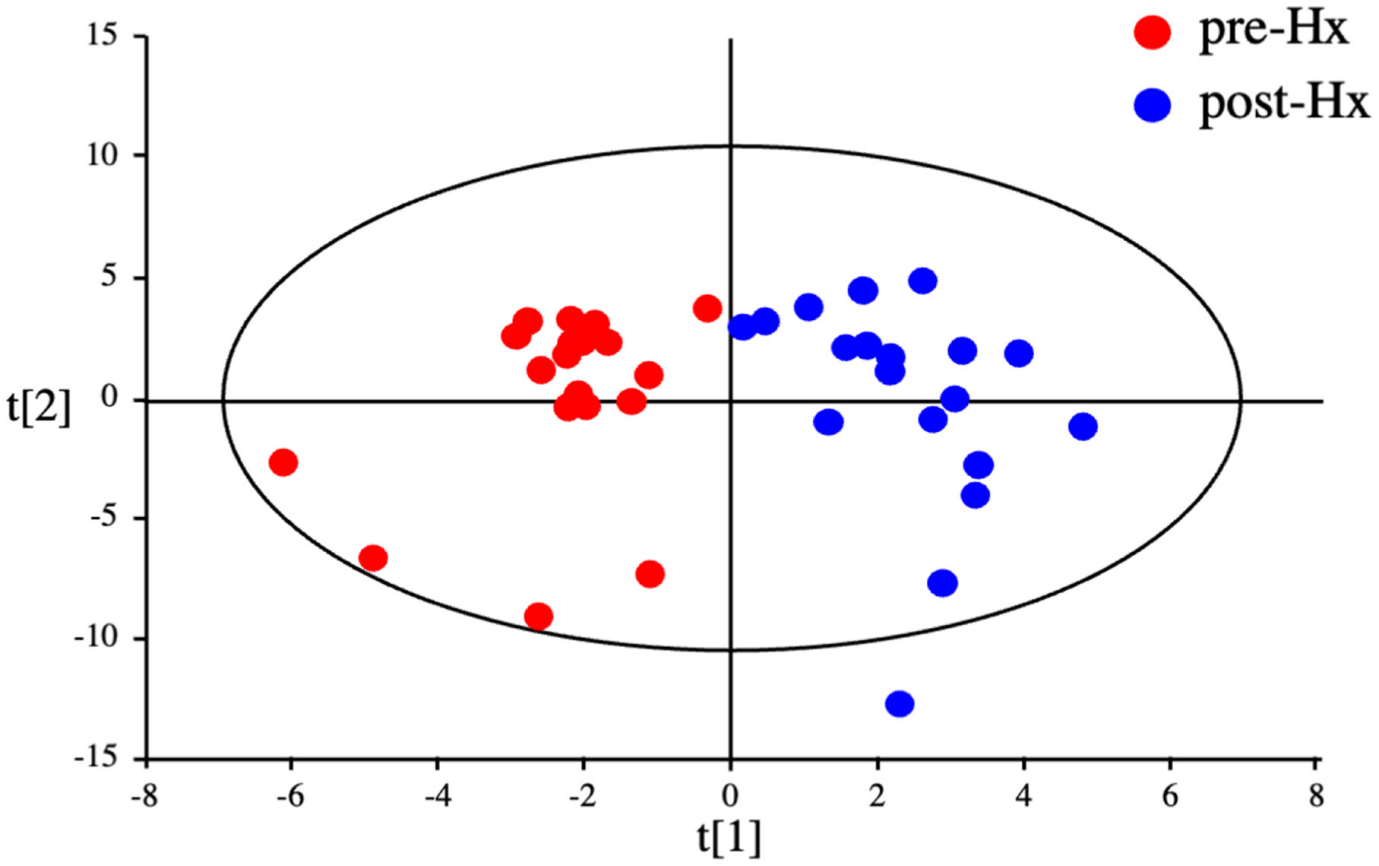
Discrimination between HCC patients before and after hepatectomy by serum metabolomics. OPLS-DA of serum metabolites from HCC patients before and after hepatectomy.

**Figure 5:**
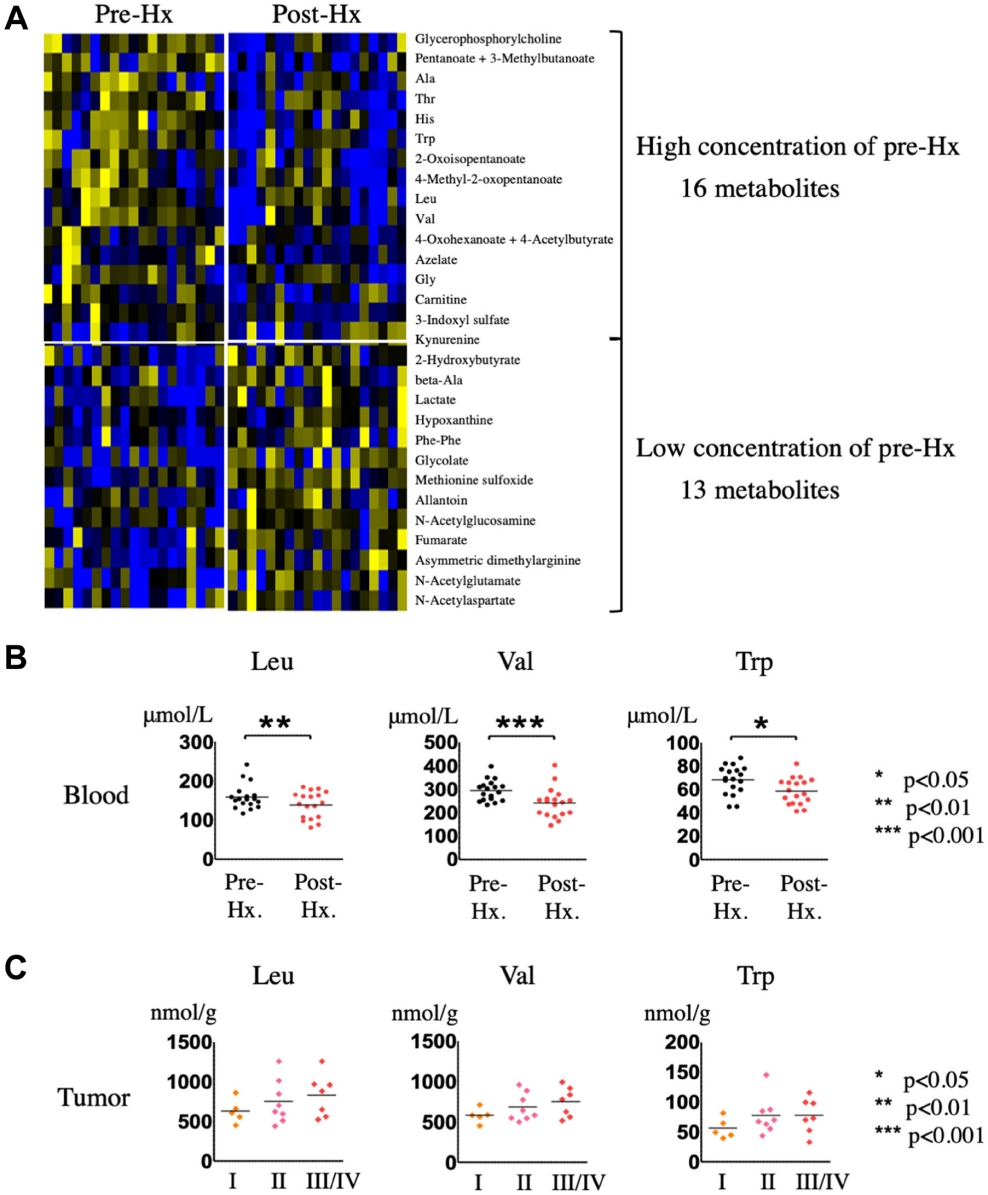
Identification and quantification of serum metabolites in HCC patients before and after hepatectomy. (**A**) Hierarchical clustering of metabolites in sera from HCC patients after hepatectomy compared with before hepatectomy. (**B**) Quantification of EAAs in sera from HCC patients after hepatectomy compared with before hepatectomy. (**C**) Quantification of EAAs in HCC tumor tissues of patients stratified according to HCC staging.

OPLS-DA statistics also showed a clear distinction between the serum metabolomes of patients before and 14 days after hepatectomy (Figure 4). Of the 110 metabolites identified, 29 had a VIP score >1.0 and a *p* value < 0.05 and were considered critical metabolites for distinguishing between pre-and post-Hx patients (Supplementary Figure 2 and Table 2). Hierarchical clustering of the metabolites indicated that serum levels of 16 metabolites increased before Hx and decreased after Hx, moreover, those of 13 metabolites decreased before Hx and increased after Hx (Figure 5A). Interestingly, 5 EAAs (leucine, valine, tryptophan, histidine, and threonine) were included in the 16 increased metabolites before Hx.

### Candidate metabolic biomarkers for hepatocarcinogenesis

To determine whether the metabolites identified here could serve as biomarkers for HCC detection, we focused on the metabolites commonly upregulated in HCC tumor compared with non-tumor tissue and increased in sera from patients pre-Hx compared with post-Hx, which included the three EAAs, leucine (VIP scores: HCC tumor tissue, 1.21722; serum, 1.36857), valine (VIP scores: HCC tumor tissue, 1.18445; serum, 2.07023), and tryptophan (VIP scores: HCC tumor tissue, 1.56405; serum, 1.70211). Figure 5B shows the serum concentrations of the three EAAs, confirming their significant increase before Hx, and significant decrease after Hx. Mean serum concentrations of leucine before and after Hx were 162 ± 32, and 139 ± 33 (μmol/L), respectively. And, mean serum concentrations of valine before and after Hx were 301 ± 51, and 242 ± 62 (μmol/L), respectively. Moreover, mean serum concentrations of tryptophan before and after Hx were 70 ± 13, and 59 ± 11 (μmol/L), respectively. We also investigated whether tumor levels of the EAAs changed as the tumor progressed, and found that leucine, valine, and tryptophan levels did indeed increase with advancing disease stage (Figure 5C). Taken together, these data suggest that leucine, valine, and tryptophan could be potential candidate metabolomic biomarkers for hepatocarcinogenesis.

## DISCUSSION

In this study, the major findings were (i) the metabolomic profile of HCC tumors differs not only compared with matched non-tumor liver tissue but also with CRLM tissue, (ii) the serum metabolome of HCC patients is altered by hepatectomy, and (iii) the three EAAs leucine, valine, and tryptophan are candidate metabolomic biomarkers for hepatocarcinogenesis. Notably, however, we found that the metabolic profiles of HCC tumor tissues of differing etiologies (HBV/HCV infection) could not be distinguished, nor could the profiles of non-tumor liver tissue from any of the patient subgroups.

Numerous genomic, transcriptomic, and proteomic studies have been performed with the goal of identifying biomarkers of hepatocarcinogenesis; however, most of the markers discovered have not yet been demonstrated to have clinical utility [27, 28]. Metabolomics could have some advantages over other omics approaches because it can comprehensively identify all low molecular weight metabolites from tissue or blood samples and can identify different phenotypes of various of diseases, including cancer [15–18]. The liver plays a central role in the metabolism of carbohydrates, amino acids, and lipids [29]; therefore, it is not surprising that normal metabolic homeostasis is easily disrupted during liver diseases such as hepatitis virus infection and non-alcoholic steatohepatitis. Metabolic reprograming of tumor cells is a well-documented phenomenon that undoubtedly provides an advantage for tumor cell survival through pathways such as energy generation, macromolecular biosynthesis, and regulation of reactive oxygen species production [24–26]. In this context, metabolomic analysis could be considered an ideal tool to identify the metabolic characteristics that discriminate between healthy subjects and HCC and between patients at different disease HCC stages. Such an ability would facilitate the discovery of reliable early diagnostic and prognostic biomarkers, as well as providing a method to monitor treatment response of patients with HCC.

Several studies have investigated HCC-associated disease signatures using metabolomics [23–26]. Xue et al. established a diagnostic signature of 13 serum metabolites (butanoic acid, ethanimidic acid, glycerol, L-isoleucine, L-valine, aminomalonic acid, D-erythrose, hexadecanoic acid, octadecanoic acid, 9,12-octadecadienoic acid, and 3 unidentified compounds) that could distinguish between HCC patients and healthy subjects [30]. Fitian et al. identified elevated serum levels of 9 kinds of metabolites, including 12-hydroxyeicosatetraenoic acid (12-HETE), 15-HETE, sphingosine, γ-glutamyl oxidative stress-associated metabolites, xanthine, serine, glycine, aspartate, and acylcarnitines, that were strongly associated with hepatocarcinogenesis in patients with HCV-associated cirrhosis [31]. Furthermore, other investigators have compared the metabolomic profiles of HCC tumor tissue and non-tumor tissue. Beyoğlu et al. classified HCC patients into six subgroups according to the presence of HBV infection, p53 mutation, and activation of Wnt/β-catenin signaling, and they found that HCC with HBV infection resulted in markedly reduced tissue concentrations of 1-stearoylglycerol, 1-palmitoylglycerol, and palmitic acid [32]. Huang et al. also demonstrated metabolic alternations associated with HCC. In that study, HCC tumor tissue contained elevated levels of glycolysis, gluconeogenesis, and β-oxidation metabolites and reduced levels of tricarboxylic acid cycle and Δ-12 desaturase metabolites compared with adjacent non-tumor tissue [33]. Thus, although several studies have investigated the general and tissue metabolomic profiles of HCC patients, the findings have varied considerably in each study.

In the present study, we identified 30 metabolites present at increased levels in HCC tumor tissue compared with non-tumor tissue, of which 10 were amino acids and 7 were EAAs. Amino acids have a variety of biological functions in energy metabolism as well as protein synthesis, and amino acid transporters thus play an important role in the growth, proliferation, and survival of both normal and cancer cells. The increase in amino acid levels observed in HCC tumor tissue may reflect the metabolic remodeling that is a hallmark of cancer cells; for example, elevation of lactate production through the Warburg effect [34] was also identified in the present study. Several investigators have demonstrated that expression and activity of amino acid transporters is increased in cancer cells, including the solute carrier protein superfamily of transporters [35] such as l-type amino acid transporters (LAT1 and LAT3), system ASC amino acid transporter, system ^X-C^ transporter-related protein, and amino acid transporter responsible for the activity of system ^B0,+^ (ATB^0,+^). These transporters have been suggested to be significantly related to tumor malignancy and carcinogenesis [36–39]. LAT1 is critical to cell growth due to its role as the main transporter of the EAAs leucine, valine, isoleucine, tryptophan, methionine, histidine, tyrosine, and phenylalanine, while LAT3 transports the EAAs leucine, valine, isoleucine, and phenylalanine, and ATB^0,+^ transports virtually the same panel of EAAs targeted by LAT1. Specifically, LAT1 has been identified as an independent prognostic factor in various cancers, including primary liver cancer, and is associated with increased tumor growth via the mammalian target of rapamycin signaling pathway [36, 38, 40, 41]. Similarly, LAT3 has been shown to upregulate the phosphoinositide 3-kinase–AKT signaling pathway via leucine transport in prostate cancer cells [42]. These amino acid transporters are likely to be highly expressed in HCC tumor tissue and may contribute to the increased EAA levels detected in our study. In future, such amino acid transporters will be investigated to elucidate the relationships for amino acids upregulation or tumor malignancy in several cancer cells.

Regarding metabolomic biomarkers for HCC diagnosis, Gao et al. have suggested that serum asparagine and β-glutamate levels may be specific biomarkers for HCC in patients with liver cirrhosis [43]. Soga et al. showed that serum γ-glutamyl dipeptides could distinguish between several liver diseases and discriminate HCC from other liver diseases, thereby serving as reliable diagnostic biomarkers for rapid screening [44]. Likewise, Saito et al. demonstrated that serum γ-glutamyl peptides are potential biomarkers for virus-related HCC [45]. Huang et al. have proposed that serum betaine and propionylcarnitine levels may have potential as complementary diagnostic markers to α-fetoprotein for HCC resulting from chronic hepatitis and cirrhosis [38]. Hence, these serum metabolic biomarkers could be useful in clinical settings in concert with serum α-fetoprotein. In the present study, we identified leucine, valine, and tryptophan as additional potential metabolomic biomarkers for HCC. Previous metabolomics studies also detected changes in serum and/or urine amino acid levels that could distinguish between patients with HCC and healthy subjects, and showed that serum levels of branched chain amino acids and aromatic amino acids are significantly higher before treatment in patients with HCC than in healthy subjects [23, 46]. However, those reports measured serum amino acids at only one time during treatment of HCC patients. In the current study, we investigated changes in the serum metabolic profiles of patients between before and after hepatectomy, and identified increases in the same EAAs that were upregulated in HCC tumor compared with non-tumor tissue. Until now, the standard value of plasma amino acids concentration has been reported in a large Japanese population with 1,890 individuals [47]. In that study, the lower and upper limits of serum concentration of leucine, valine, and tryptophan were revealed as follows; leucine 76.7 to 159.5, valine: 143.0 to 287.0, and tryptophan: 42.9 to 74.4 μmol/L. In the current study, serum concentration of leucine and valine before Hx exceeded each upper limit and that of tryptophan was almost equivalent to its upper limit. Besides, serum concentrations of those three EEAs decreased within normal range after Hx. Therefore, our results suggest that measurement of serum leucine, valine, and tryptophan levels could facilitate the early diagnosis of HCC and also be used as a reliable metabolomic biomarker to monitor disease progression and treatment response.

The current and previous findings provide a rationale for further investigations into the potential utility of leucine, valine, and tryptophan as diagnostic and prognostic biomarkers for HCC. However, our study has several limitations. First, the sample size was small and may not be generalizable. Second, selection bias was possible, because of the retrospective nature of the study. Third, we did not confirm our findings in a second validation set of samples. Finally, we indicated did not prove the role of those three EEAs for hepatocarcinogenesis and tumor malignancy, with *in* *vitro* or *in vivo* study. These issues should be addressed by additional studies to clarify the potential for the three EAAs to serve as reliable metabolomic biomarkers of HCC.

## MATERIALS AND METHODS

### Patient selection and sample collection

We enrolled 20 patients with HCC without detectable distant organ metastases who underwent hepatectomy at Tokushima University Hospital, Japan between December 2013 and February 2015. The patients consisted of 16 men and 4 women and ranged in age from 44 years to 78 years (mean 65.5 years) (Supplementary Table 1). An additional cohort of 4 patients with liver metastasis from colorectal cancer (CRLM) were included as a comparison group for the analyses. All 24 patients underwent liver resection as their initial treatment without preoperative chemotherapy or radiotherapy, and curative resection was confirmed by pathological examinations. Also, those included patients did not receive any amino acid preparation during the perioperative period. We excluded patients with intrahepatic cholangiocarcinoma, such as mixed-type liver cancer. Tumor status and stage were defined according to the Classification of Primary Liver Cancer by the Liver Cancer Study Group of Japan [48]. Age, sex, liver function, hepatitis virus infection status, and tumor pathological status were recorded. Tumor and non-tumor tissue samples were obtained immediately after hepatectomy. Blood samples were taken immediately before (pre-Hx) and 14 days after (post-Hx) hepatectomy. The samples were snap frozen at −80°C and stored until analysis. The study protocol was approved by the Institutional Review Board of the University of Tokushima Graduate School (approval no. 2274-1), and all patients provided written informed consent.

### Metabolome analysis

Frozen tissue (approximately 40 mg per sample) was added to methanol (500 μL) containing internal standards (20 μmol L^−1^ each of methionine sulfone and D-camphor-10-sulfonic acid) and homogenized using a bead beater (TOMY Micro Smash MS-100R; Tomy Digital Biology, Tokyo, Japan) at 3000 rpm for 60 s. Chloroform (500 μL) and Milli-Q water (200 μL) were added to the homogenate, which was thoroughly mixed and then centrifuged at 4600 *g* for 15 min at 4°C. After centrifugation, the aqueous fraction was ultrafiltered using a 5 kDa cutoff ultra-centrifugal filter unit (Ultrafree-MC-PLHCC-HMT; Human Metabolome Technologies Inc., Tsuruoka, Japan). The filtrate was dried using a vacuum centrifuge and dissolved in Milli-Q water (50 μL) containing 200 μmol L^-1^ of the reference compounds (3-aminopyrrolidine and trimesic acid) prior to CE-TOFMS analysis. Serum samples were obtained by centrifugation of blood samples at 3000 *g* at 4°C for 10 min. The samples were divided into aliquots and stored at −80°C until extraction of metabolites. CE-TOFMS-based metabolomic profiling and data analysis were carried out essentially as described [21, 49–52].

### Statistical analysis

Statistical analyses were carried out using Prism 6.07 for Windows (GraphPad Software Inc., La Jolla, CA, USA). A *p* value of < 0.05 was considered statistically significant, and data were presented with mean ± standard deviation (SD). The variable importance in the projection (VIP) obtained from multivariate statistical analysis can provide significantly changed variables of surgical specimen between HCC tumor and non-tumor tissue, and serum sample between pre-Hx and post-Hx. HCC tumor and non-tumor tissue value and Pre-Hx and post-Hx values were compared using the Wilcoxon matched-pairs signed-rank test. Heat maps of metabolite levels were generated using hierarchical clustering based on Pearson’s correlation coefficients with Multi Experiment Viewer software (Institute for Genomic Research, Rockville, MD, USA). The data were exported and analyzed by orthogonal partial least squares discriminant analysis (OPLS-DA) and principal component analysis (PCA) using SIMCA-P software 12.0.1 (Umetrics AB, Umea, Sweden) to visualize the metabolic changes between HCC tumor and non-tumor tissue or Pre-Hx and post-Hx after mean centering and unit variance scaling.

## SUPPLEMENTARY MATERIALS



## Abbreviations

HCC: hepatocellular carcinoma
HBV: hepatitis B virus
HCV: hepatitis C virus
CE-TOFMS: capillary electrophoresis-time-of-flight mass spectrometry
CRLM: colorectal liver metastasis
OPLS-DA: orthogonal partial least squares discriminant analysis
PCA: principal component analysis
VIP: variable importance for projection
EAA: essential amino acid
LAT: l-type amino acid transporter
ATB^0,+^: amino acid transporter responsible for the activity of system ^B0,+^
Hx: hepatectomy
ICGR15: indocyanine green retention 15
AFP: alpha-fetoprotein
DCP: Des-gamma-carboxy prothrombin
