# Supplementary material for: Essential amino acids as diagnostic biomarkers of hepatocellular carcinoma based on metabolic analysis
Source: Oncotarget. 2022 Nov 22;13:1286–98. doi: 10.18632/oncotarget.28306 (PMC11623405; doi:10.18632/oncotarget.28306)
Supplement: Supplementary file 1 [file oncotarget-13-28306-s001.pdf]

Essential amino acids as diagnostic biomarkers of hepatocellular carcinoma based on metabolic analysis

SUPPLEMENTARY MATERIALS

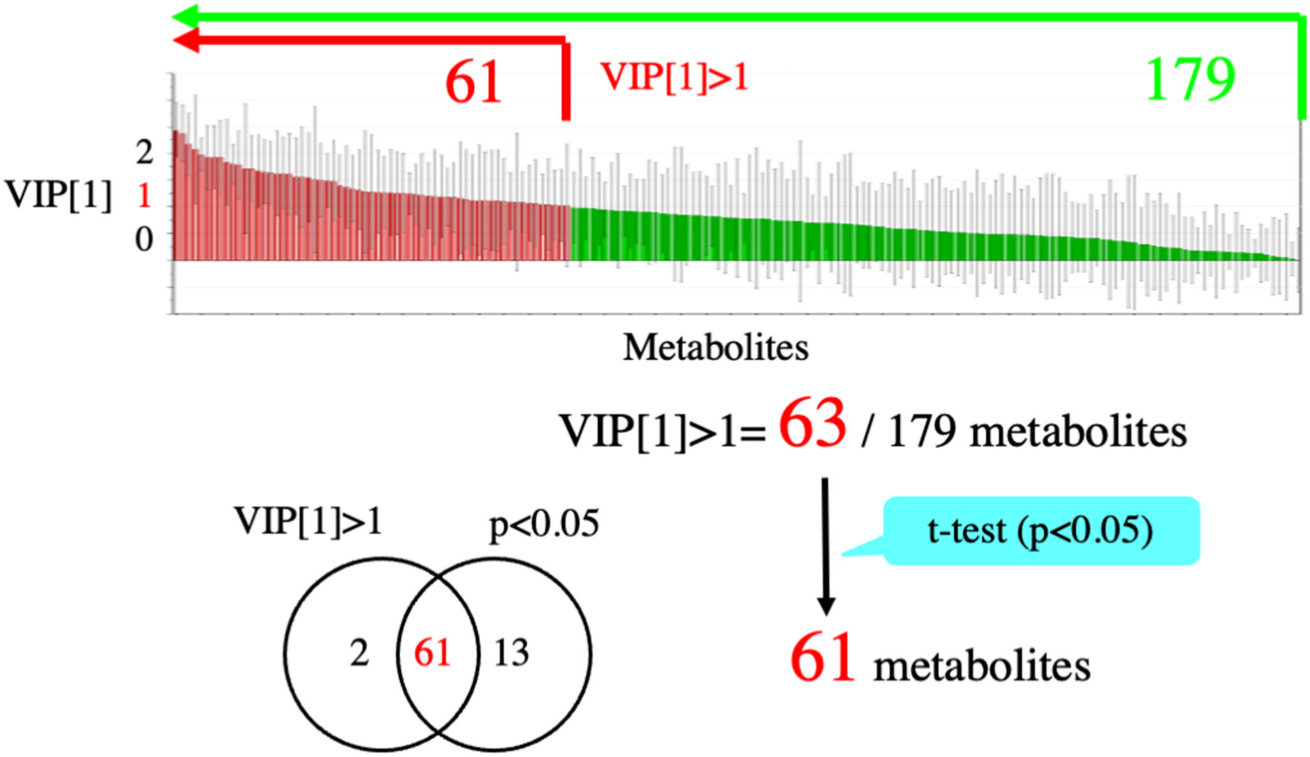

Supplementary Figure 1: Selection of key metabolites that discriminate between HCC tumor and non-tumor tissues Of the 179 metabolites detected in HCC tumor tissue, 61 had a VIP score >1.0 at $p < 0.05$ and were selected as critical metabolites for discriminating between tumor and non-tumor tissue.

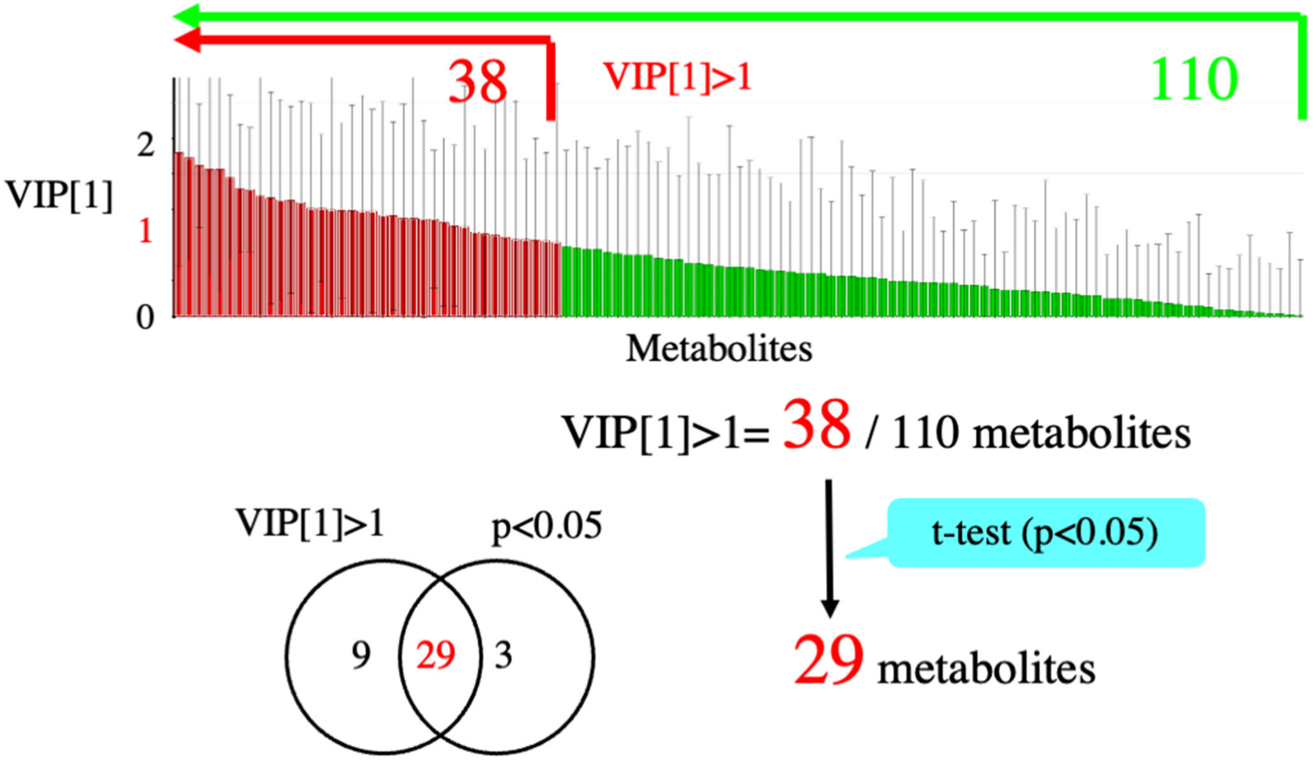

Supplementary Figure 2: Selection of key serum metabolites that discriminate between HCC patients before and after hepatectomy Of the 110 metabolites detected in serum samples from HCC patients after hepatectomy, 29 had a VIP score >1.0 at p < 0.05 and were selected as critical metabolites for discriminating between patients before and after hepatectomy.

Supplementary Table 1: Clinicopathological characteristics of the 20 patients with HCC

Factors		
Age (years)	Median ± SD	65.5 ± 8.0
Gender	Male/Female	16/4
Diabetes mellitus	Absence/Presence	13/7
Hepatic viral infection	Negative/HBV/HCV	7/6/7
ICG R15 (%)	≦10/>10	12/8
Stage	I, II/III, IV	13/7
Maximum tumor size	<5 cm/≥5 cm	13/7
Number	Single/Multiple	16/4
Growth type	Expandable/Invasive	10/10
Differentiation	Well~mod/Others	18/2
Portal invasion	Negative/Positive	14/6
Venous invasion	Negative/Positive	20/0
AFP (ng/ml)	≦100/>100	12/8
DCP (IU/L)	≦300/>300	11/9

Abbreviations: ICGR15: indocyanine green retention 15; HBV: hepatitis B virus; HCV: hepatitis C virus; AFP: alpha-fetoprotein; DCP: Des-gamma-carboxy prothrombin.
